# Differential Effects of Inhibitor Combinations on Lysophosphatidic Acid-Mediated Chemokine Secretion in Unprimed and Tumor Necrosis Factor-α-Primed Synovial Fibroblasts

**DOI:** 10.3389/fphar.2017.00848

**Published:** 2017-11-21

**Authors:** Weili Hui, Chenqi Zhao, Sylvain G. Bourgoin

**Affiliations:** ^1^Division of Infectious Disease and Immunity, Centre Hospitalier Universitaire de Québec Research Center, Quebec City, QC, Canada; ^2^Faculty of Medicine, Laval University, Quebec City, QC, Canada

**Keywords:** lysophosphatidic acid, tumor necrosis factor-α, MSK, ERK, p38MAPK, CREB Fibroblast-like synoviocytes

## Abstract

Lysophosphatidic acid (LPA) is a pleiotropic bioactive lysophospholipid involved in inflammatory mediator synthesis. Signaling through p38MAPK, ERK, Rho kinase, and MSK-CREB contributes to LPA-mediated IL-8 production in fibroblast-like synoviocytes (FLS) from rheumatoid arthritis (RA) patients. The study was undertaken to investigate how LPA activates MSKs and how signaling crosstalk between TNFα and LPA contributes to the super-production of cytokines/chemokines. RAFLS pretreated or not with TNFα were stimulated with LPA. Immunoblotting with phospho-antibodies monitored MSK activation. Cytokine/chemokine production was measured using ELISA and multiplex immunoassays. LPA induced MSK activation by signaling through ERK whereas p38MAPK, Rho kinase, NF-κB or PI3K contribute to IL-8 synthesis mainly via MSK-independent pathways. Priming with TNFα enhanced LPA-mediated MSK phosphorylation and cytokine/chemokine production. After priming with TNFα, inhibition of ERK or MSK failed to attenuate LPA-mediated IL-8 synthesis even if the MSK-CREB signaling axis was completely or partially inhibited. In TNFα-primed cells, inhibition of LPA-mediated cytokine/chemokine synthesis required a specific combination of inhibitors such as p38MAPK and ERK for IL-8 and IL-6, and Rho kinase and NF-κB for MCP-1. The ability of the signaling inhibitors to block LPA induced cytokine/chemokine synthesis is dependent on the inflammatory cytokinic environment. In TNFα-primed RAFLS the super-production of IL-8 and IL-6 induced by LPA occurs mainly via MSK-independent pathways, and simultaneous inhibition of at least two MAPK signaling pathways was required to block their synthesis. Since simultaneous inhibition of both the p38MAPK and ERK-MSK-CREB pathways are required to significantly reduce LPA-mediated IL-8 and IL-6 production in TNFα-preconditioned RAFLS, drug combinations targeting these two pathways are potential new strategies to treat rheumatoid arthritis.

## Introduction

Rheumatoid arthritis is a systemic, severe autoimmune disease associated with chronic inflammation of peripheral joints and adjacent tissues, as well as hyperplasia of synovial lining cells (synovial fibroblasts) along with infiltration of immune cells into the synovial cavity, forming a pannus, leading to joint deformation and pain (Bartok and Firestein, 2010). Synovial fibroblasts contribute to inflammation by secreting various cytokines/chemokines and matrix metalloproteases in response to inflammatory mediators such as tumor necrosis factor alpha (TNFα) and LPA (Bourgoin and Zhao, 2010; Lee et al., 2013).

Lysophosphatidic acid is a monoacyl phospholipid acting as an extracellular molecule involved in many physiological and pathophysiological conditions (Yung et al., 2014). LPA can be produced from LPC by ATX (Moolenaar and Perrakis, 2011). The presence of ATX, LPC and LPA has been detected in synovial fluids from RA patients (Fuchs et al., 2005; Nochi et al., 2008; Zhao et al., 2008; Nikitopoulou et al., 2012; Miyabe et al., 2013). An average of 3.7 ± 2.2 μM LPA was reported in synovial fluids form RA patients (Nochi et al., 2008). LPA signals via binding to its G-protein-coupled receptors, which in turn trigger various downstream signaling cascades through activation of associated heterotrimeric G proteins, including Gi/o, G12/13, Gq, and Gs (Yung et al., 2014). Six LPA receptors named LPA_1_-LPA_6_ have been identified (Kihara et al., 2014). Synovial fibroblasts express LPA_1_, LPA_2_ and LPA_3_, of which LPA_1_ is the most abundant (Zhao et al., 2008; Bourgoin and Zhao, 2010). Genetic deletion of LPA_1_ in mice conferred resistance to type II collagen-induced arthritis (Miyabe et al., 2013). Treatment of mice with an LPA_1_ antagonist (Miyabe et al., 2013; Orosa et al., 2014) or genetic ablation of ATX in synovial fibroblasts (Nikitopoulou et al., 2012) reduced the severity of arthritis.

LPA_1_ and LPA_3_ are both involved in LPA-mediated cytokine/chemokine release by RAFLS *in vitro* (Zhao et al., 2008), and using the murine air pouch model (Zhao et al., 2011). LPA_1_ also mediates synovial fibroblast migration (Bourgoin and Zhao, 2010) and confers resistance to TNFα-induced apoptosis (Orosa et al., 2012). The signaling pathways activated by LPA have been reported to include extracellular-signal-regulated kinase (ERK), mitogen activated protein kinase (p38MAPK), and Rho kinase (ROCK) (Zhao et al., 2008).

Mitogen- and stress-activated protein kinases 1 and 2 (MSKs, formerly called ribosomal protein S6 kinases A5 and A4) can be activated by either ERK or p38MAPK (Arthur, 2008; Vermeulen et al., 2009). MSK1 is phosphorylated on multiple sites including Ser-360, Thr-581, Thr-700, Ser-212, Ser-376, Ser-381, Thr-630, Ser-647, Ser-657, and Ser-695 in response to various agonists (McCoy et al., 2007). MSK1 is first phosphorylated by ERK and p38MAPK at Ser-360, Thr-581, and Thr-700 (Deak et al., 1998; McCoy et al., 2007). This causes activation of the C-terminal kinase domain of MSK1, which leads to autophosphorylation of Ser-212, Ser-376 and Ser-381 (McCoy et al., 2005, 2007). Phosphorylation of Ser-212 and Ser-376 are essential for activation of the MSK1 N-terminal kinase domain (McCoy et al., 2005, 2007). MSK1 and MSK2 are nuclear proteins that regulate the expression of several immediate-early genes through phosphorylation of transcription factors including CREB, ATF-1, p65 and STAT3, as well as chromatin components such as histone H3 and HMGN1 (Arthur, 2008; Vermeulen et al., 2009; Reyskens and Arthur, 2016). The MSK-CREB signaling pathway is activated by LPA and contributes to cytokine/chemokine production in RAFLS (Zhao et al., 2014).

TNFα and IL-6 are key components in the cytokine network of RA (Srirangan and Choy, 2010; McInnes et al., 2016). IL-8, MCP-1/CCL2, RANTES/CCL5 and IP-10 also contribute to the pathogenesis of RA as chemotactic factors of neutrophils (Bickel, 1993), monocytes (Stankovic et al., 2009) or T cells (Pavkova Goldbergova et al., 2012; Antonelli et al., 2014). Previous study showed that induction of a pro-inflammatory environment by TNFα upregulates LPA_3_ expression and strongly enhances cytokine/chemokine release induced by LPA (Zhao et al., 2008). LPA_1_ largely contributes to LPA-mediated chemokine synthesis such as IL-6 (Miyabe et al., 2014). However, silencing of LPA_1_ was reported to increase chemokine/cytokine synthesis in response to TNFα possibly through increased activation of the MAPK pathways (Orosa et al., 2012).

In the present study we extensively studied how the multiple signaling pathways that contribute to LPA-induced chemokine/cytokine super-production in TNFα-primed RAFLS are associated with increased signaling through the MSK-CREB axis. We confirmed that inhibition of p38MAPK or ERK alone can reduce LPA-induced cytokine/chemokine secretion, and showed in TNFα-primed RAFLS that inhibition of both p38MAPK or ERK is critical to reduce MSK-CREB signaling and specifically inhibits IL-6 and IL-8 synthesis induced by LPA. This study provides insight into the mechanism whereby signaling crosstalk between LPA and TNFα results in synergistic induction of cytokine/chemokine secretion in RAFLS.

## Materials and Methods

### Reagents

TNFα was purchased from PeproTech Inc. (Rocky Hill, NJ, United States). 1-Oleoyl-sn-glycerol 3-phosphate sodium salt (LPA, 18:1) was purchased from Sigma-Aldrich Canada (Oakville, ON, Canada). Antibodies against human phospho-MSK1 (Ser-376)/MSK2 (Ser-360), phospho-MSK1 (Ser-212), MSK2 and GAPDH were from R&D Systems Inc. (Minneapolis, MN, United States). Antibodies against human phospho-CREB (Ser-133), phospho-MSK1 (Ser-360), phospho-MSK1 (Thr-581) and MSK1 were purchased from Cell Signaling Technology (Beverly, MA, United States). Antibody to actin was from Sigma–Aldrich Canada (Oakville, ON, Canada). Inhibitors of p38MAPK (SB203580), ERK (PD98059), Rho kinase (Y27632), and NF-κB (Bay11-7082) were purchased from Calbiochem (San Diego, CA, United States). The PI3K inhibitor wortmannin was from Millipore Corporation (St. Charles, MO, United States). MSK inhibitor SB-747651A was obtained from Axon Medchem (Groningen, The Netherlands). Human IL-8 ELISA kit was purchased from BioSource International Inc. (Camarillo, CA, United States). The human cytokine/chemokine Luminex multiplex immunoassay kit (Milliplex^®^ MAP Kit, detecting MCP-1, IL-6, IL-8, IP-10, and RANTES) was from Millipore Corporation (St. Charles, MO, United States). FBS and DMEM were from Wisent Inc. (St-Bruno, QC, Canada). Propidium iodide and Annexin V-eFluor450 were from BD Pharmingen (Oakville, ON, Canada).

### Cell Culture and Treatment

Human primary FLS were obtained from RA patients who were diagnosed according to the criteria developed by the American College of Rheumatology and were undergoing joint surgery (Arnett et al., 1988). Human primary FLS at passage 0 or 1 were purchased from Asterand (Detroit, MI, United States) or isolated from RA synovial membrane specimens collected from RA patients, after informed and written consent was obtained and with the approval of the CHU de Québec-Laval University ethics committee (B14-04-1946). FLS were isolated from synovial membrane specimens as described previously (Faour et al., 2003; Zhao et al., 2008). Cells were cultured under standard conditions (37°C and 5% CO_2_) and grown in DMEM supplemented with 10% FBS, penicillin (100 IU), and streptomycin (100 M) as described previously (Zhao et al., 2008, 2014). Cells from three different donors were miscellaneously used in all experiments at passages 2–7.

For the experiments, semi-confluent RAFLS were starved in FBS-free DMEM for 24 h. To evaluate the effect of TNFα, starved cells were pre-incubated with TNFα (80 ng/ml) for 8 h prior to LPA treatment (Supplementary Figure 1). Where indicated, cells were pre-treated with the inhibitors of p38MAPK (SB203580), ERK (PD98059), Rho kinase (Y27632), PI3K (wortmannin), NF-κB (Bay11-7082), and MSK (SB-747651A) at indicated concentrations for 30 min as described previously (Zhao et al., 2008, 2014). In some experiments combinations of signaling inhibitors were also used. Cells were then washed with serum-free DMEM and stimulated with 5 μM LPA in fresh serum-free DMEM containing testing compounds (Supplementary Figure 1). For measurement of cytokine/chemokine synthesis cells were stimulated for 24 h as described previously (Zhao et al., 2008, 2014). Cell culture supernatants were collected and stored at -80°C until the ELISA and the multiplex immunoassay for human cytokines/chemokines were performed. Data points in each of the independent experiments were performed in duplicate. To monitor the levels of MSK phosphorylation RALFS were stimulated with 5 μM LPA for 5 min, a time at which phosphorylation of MSK1 at Ser-376 or of MSK2 at Ser-360 is maximal (Zhao et al., 2014).

### Analyses of Cytokine/Chemokine Synthesis

IL-8 was monitored using ELISA according to the manufacturer’s protocol. Optical densities were determined using a SoftMaxPro5 plate reader at 450 nm. The detection range of the IL-8 ELISA kit is 15.6-1000 pg/ml.

Human cytokines/chemokines IL-6, IL-8, IP-10, MCP-1, and RANTES were monitored using a Luminex multiplex immunoassay (Milliplex^®^ MAP Kit) according to the manufacturer’s instructions. The dynamic range of the assay is 3.2-10000 pg/ml. The assay sensitivities (minimum detectable concentrations, mean ± SD, pg/ml) were 0.9 ± 1.3, 0.4 ± 0.7, 8.6 ± 14, 1.9 ± 3.4, and 1.2 ± 1.9 for IL-6, IL-8, IP-10, MCP-1, and RANTES, respectively.

### Analyses of MSK and CREB Phosphorylation

RAFLS were lysed in boiling sample buffer [50 mM Tris-HCl (pH6.8), 10% (v/v) glycerol, 50 mM DTT, 4% (v/v) SDS] for 7–10 min. Equal amounts of protein were loaded on gels and separated by 10% SDS-polyacrylamide gel electrophoresis. Proteins were later transferred from polyacrylamide gel to methanol-soaked PVDF membranes (Pall Canada Ltd., Ville St-Laurent, QC, Canada). Primary antibody incubation was performed overnight at 4°C, 1 h at 37°C, or 2 h at room temperature according to optimization tests. The membranes were washed three times and incubated with appropriate horseradish peroxidase-conjugated secondary antibodies at room temperature for 1 h. Membranes were when washed three times and antibody-antigen complexes were revealed using Western Lightning chemiluminescence reagent according to the manufacturer’s instructions (Perkin Elmer Life Sciences, Woodbridge, ON, Canada).

### Statistical Analysis

Unless otherwise stated, the data are from at least three independent experiments presented as mean values ± SEM. Prism 7.0 software was used for all statistical analyses. Statistical significance of the difference between samples of two different treatments was determined by *t*-test (two-tailed *p*-value). For studies using samples from more than one treatment, statistical significance between control and treated cells was determined by one-way ANOVA or two-way ANOVA multiple comparison test according to the context. P values less than 0.05 were considered statistically significant.

## Results

### TNFα Up-Regulates MSK and CREB Phosphorylation Induced by LPA in RAFLS

**FIGURE 1:**
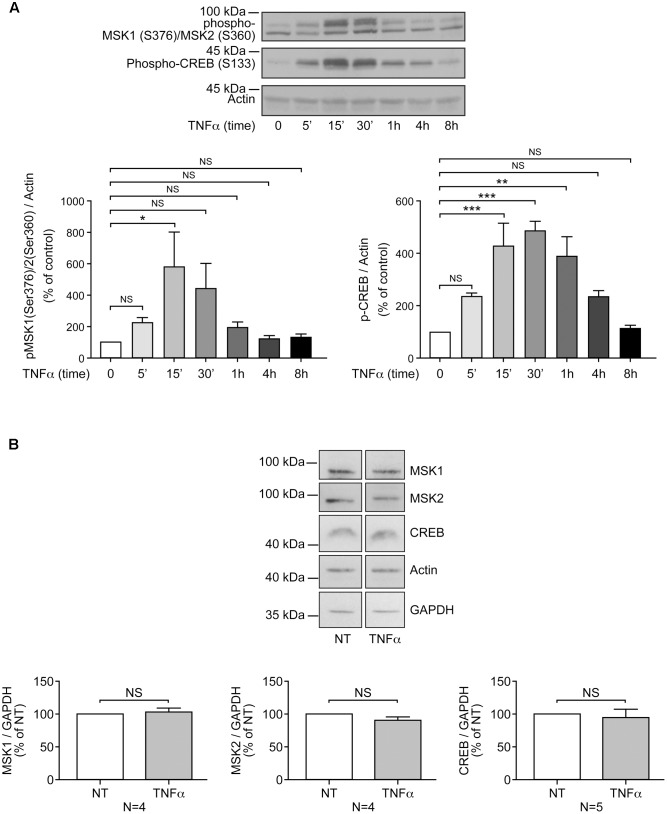
Priming of RAFLS with TNFα transiently enhances MSK phosphorylation and CREB phosphorylation, but not MSKs or CREB protein expression. RAFLS were treated with TNFα (80 ng/ml) for indicated time points. Cell lysates were subjected to Western blot analyses with the indicated antibodies. **(A)** Kinetics of MSK1/MSK2 and CREB phosphorylation in response to stimulation with TNFα. The upper panel is a Western blot representative of three independent experiments and lower panels are the densitometry quantification analysis. Only the upper band recognized by the p-MSK1/2 antibody was scanned and normalized to actin. **(B)** Priming of RAFLS with TNFα for 8 h did not affect the expression of MSKs or CREB. Upper panels are Western blots representative of at least three independent experiments and lower panels are the densitometry quantification analysis. Depending on the protein molecular weight data were normalized with respect to actin or GAPDH as a loading control. The basal phospho-protein levels in unstimulated RAFLS were set to 100%. Data are the mean value ± SEM. The values in A and B were subjected to a one-way ANOVA, Dunnett’s multiple comparison test and *T*-test, respectively. ^∗^*p* < 0.05; ^∗∗^*p* < 0.01; ^∗∗∗^*p* < 0.001.

**FIGURE 2:**
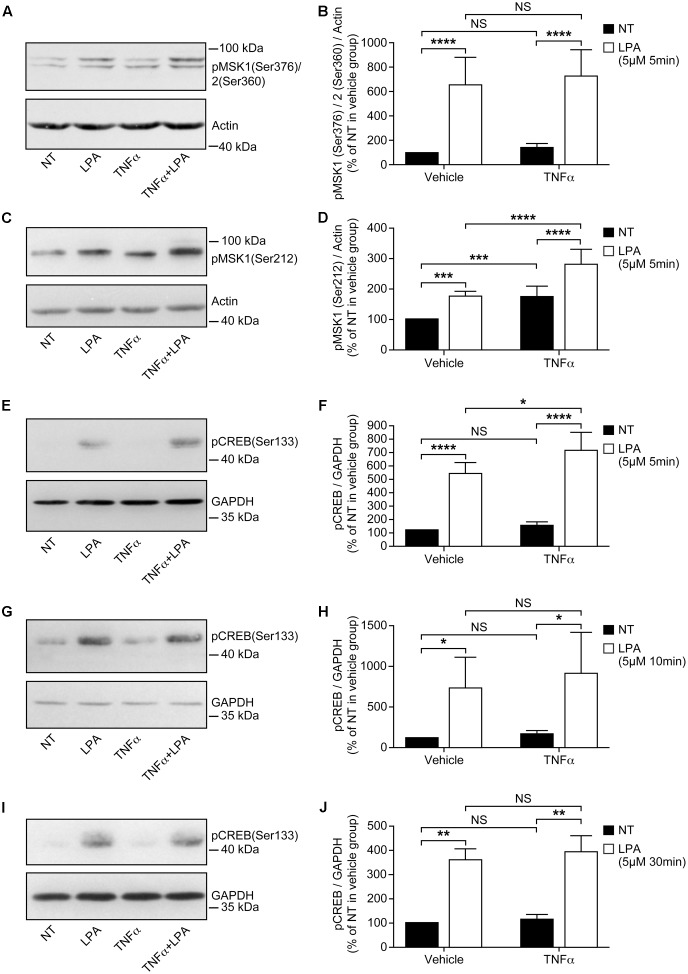
Priming of RAFLS with TNFα enhances LPA-mediated MSK phosphorylation and CREB phosphorylation. RAFLS were treated with or without TNFα (80 ng/ml) for 8 h before stimulation with 5 μM LPA for 5 min **(A–F)**, 10 min **(G,H)**, and 30 min **(I,J)**. Cell lysates were subjected to Western blot analyses with the indicated antibodies. The left **(A,C,E,G,I)** are Western blots representative of independent experiments with similar results. The right (**B**; *n* = 22), (**D**; *n* = 17), (F; *n* = 10), (H; *n* = 3), and (J; *n* = 4) are the densitometry quantification analysis. In panel A only the upper band recognized by the p-MSK1/2 antibody was scanned. Data were normalized with respect to actin or GAPDH as a loading control. The non-treated sample (NT) was set to 100% for comparison between experiments. Data are the mean value ± SEM. The values were subjected to a two-way ANOVA, Sidak’s multiple comparison test. ^∗^*p* < 0.05; ^∗∗^*p* < 0.01; ^∗∗∗^*p* < 0.001; ^∗∗∗∗^*p* < 0.0001.

Our previous study has shown the key role of the MSK-CREB signaling axis in LPA-mediated IL-8 secretion in RAFLS (Zhao et al., 2014). LPA was reported to induce a transient phosphorylation of MSK1 at Ser-376 and/or MSK2 at Ser-360 (p-MSK1/2), as well as phosphorylation of CREB at Ser-133 (Zhao et al., 2014). Since cell preconditioning with TNFα synergistically enhances LPA-mediated cytokine/chemokine synthesis (Zhao et al., 2008), we first examined the effect of TNFα on the kinetics of MSK1/2 and CREB phosphorylation. TNFα also induced a transient increase in p-MSK1/2 that was maximal at 15 min (∼6-fold increase) and returned to near basal levels by 4 h (**Figures 1A**, **2A,B**). After 8 h stimulation with TNFα phosphorylation of MSK1 at Ser-212 (p-MSK1) remained significantly elevated (80 ± 32%, *p* < 0.001) when compared to the non-treated cells (**Figures 2C,D**). Further efforts to detect MSK1 phosphorylation at Ser-360 or Thr-581 were fruitless due to the detection threshold of the antibodies and of the chemiluminescence reagent used.

Stimulation of RAFLS with LPA for 5 min led to a six-fold increase in the levels of p-MSK1/2 (**Figures 2A,B**). LPA stimulation for 5 min after treatment with TNFα for 8 h did not increase the levels of p-MSK1/2 (**Figures 2A,B**). The levels of p-MSK1 were increased by ∼2-fold in response to LPA stimulation for 5 min. (**Figures 2C,D**). TNFα treatment for 8 h significantly enhanced the levels of p-MSK1 induced by LPA (**Figures 2C,D**). The levels of p-MSK1 were increased by 56 ± 16% (*p* < 0.0001) when compared to LPA-treated samples.

CREB phosphorylation in response to TNFα was maximal at 30 min (∼4.5-fold increase) and slowly declined to baseline levels by 8 h (**Figure 1A**). In all subsequent experiments with TNFα the time point 8 h, when p-CREB returned to baseline levels, was selected to monitor LPA-induced signaling and functional responses in primed cells. RAFLS stimulation with LPA for 5 min led to a ∼5-fold increase in the levels of p-CREB (**Figures 2E,F**). TNFα pretreatment for 8 h significantly enhanced LPA-mediated CREB phosphorylation at 5 min but not at the later time points tested (**Figures 2E–J**).

Overall the data suggest that TNFα priming for 8 h tends to increase LPA-mediated phosphorylation of MSK1/2 and CREB. Since TNFα has no effect on MSK1, MSK2, and CBEB protein expression (**Figure 1B**), the data would suggest that increased signaling through the MSK1/2-CREB axis may in turn enhance LPA-induced responses in TNFα-primed RAFLS.

### Signaling Pathways Involved in LPA-Mediated MSK Phosphorylation

Previous studies showed that signaling through ERK, p38MAPK, Rho kinase, and MSK-CREB regulates LPA-induced cytokine/chemokine secretion (Zhao et al., 2008, 2014). In addition to Rho kinase, ERK and p38MAPK, PI3K was also reported to be involved in LPA-mediated IL-6 secretion (Chou et al., 2005) and cell migration (Sengupta et al., 2003; Du et al., 2010; Riaz et al., 2016). MSKs are phosphorylated by ERK and p38MAPK (Arthur, 2008; Vermeulen et al., 2009). To test whether ERK and p38MAPK signal via MSKs in our model, we used kinase-selective inhibitors and first monitored the levels of p-MSK1/2 and p-MSK1 as a read out.

**FIGURE 3:**
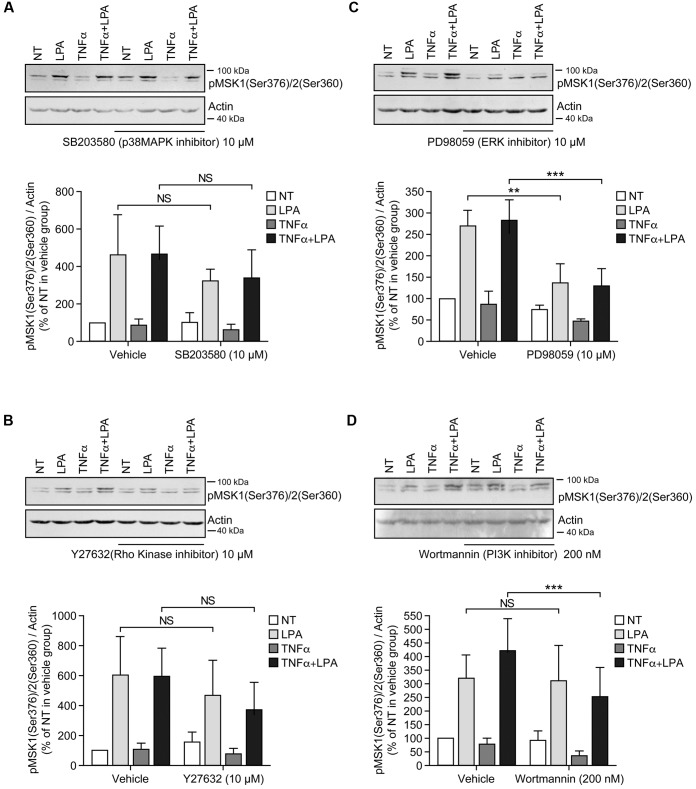
Inhibition of ERK1/2 blocks LPA-mediated MSK1 Ser-376/MSK2 Ser-360 phosphorylation in TNFα-primed RAFLS. RAFLS were treated with or without TNFα (80 ng/ml) for 8 h before stimulation with 5 μM LPA for 5 min. Where indicated the cells were pre-treated for 30 min with the inhibitors prior to stimulation with LPA. The levels of p-MSK1 Ser-376/MSK2 Ser-360 were monitored as described in “Materials and Methods.” Upper panels are Western blots representative of 4 **(A,C)** or 6 **(B,D)** independent experiments with similar results. Lower panels are the densitometry quantification of p-MSK1 Ser376/MSK2 Ser-360. Only the upper band recognized by the p-MSK1/2 antibody was scanned and normalized with respect to actin as a loading control. The non-treated sample (NT) was set to 100% for comparison between experiments. Data are the mean value ± SEM. The resulting values were subjected to a two-way ANOVA, Sidak’s multiple comparison test. ^∗∗^*p* < 0.01; ^∗∗∗^*p* < 0.001.

**FIGURE 4:**
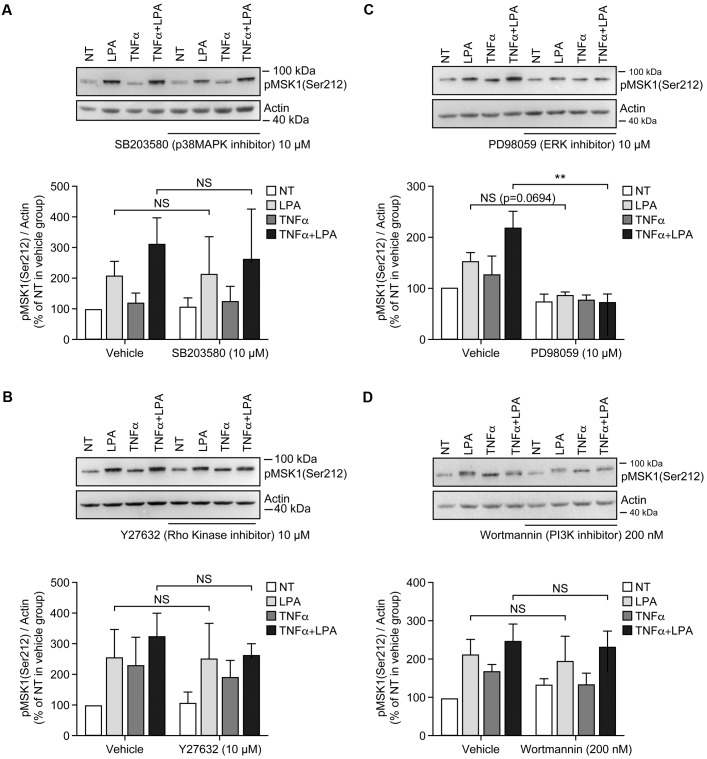
Inhibition of ERK1/2 blocks LPA-mediated MSK1 Ser-212 phosphorylation in TNFα-primed RAFLS. The cells were pre-treated with the inhibitor of p38MAPK **(A)**, Rho kinase **(B)**, ERK **(C)**, and PI3K **(D)** for 30 min prior to stimulation with LPA. The samples were analyzed exactly as described in **Figure 3**, except that p-MSK1 Ser-212 was monitored. Data are the mean value ± SEM. The values were subjected to a two-way ANOVA, Sidak’s multiple comparison test. ^∗∗^*p* < 0.01.

Incubation of RAFLS with inhibitors of p38MAPK, Rho kinase, or PI3K did not affect the level of p-MSK1/2 or p-MSK1 induced by LPA (**Figures 3A,B,D**, **4A,B,D**). In contrast, the ERK inhibitor significantly decreased the levels of p-MSK1/2 (50 ± 20%, *p* < 0.01) (**Figure 3C**), and reduced that of p-MSK1 (42 ± 7%, *p* = 0.0694) induced by LPA (**Figure 4C**).

Inhibition of ERK activation after priming with TNFα for 8 h significantly reduced the levels of p-MSK1/2 and p-MSK1 induced by LPA by 54 ± 18% (**Figure 3C**) and 66 ± 8% (**Figure 4C**), respectively. Inhibition of PI3K in TNFα-primed RAFLS stimulated with LPA significantly decreased the levels of p-MSK1/2 by 48 ± 10% (**Figure 3D**) but did not affect those of p-MSK1 (**Figure 4D**).

**FIGURE 5:**
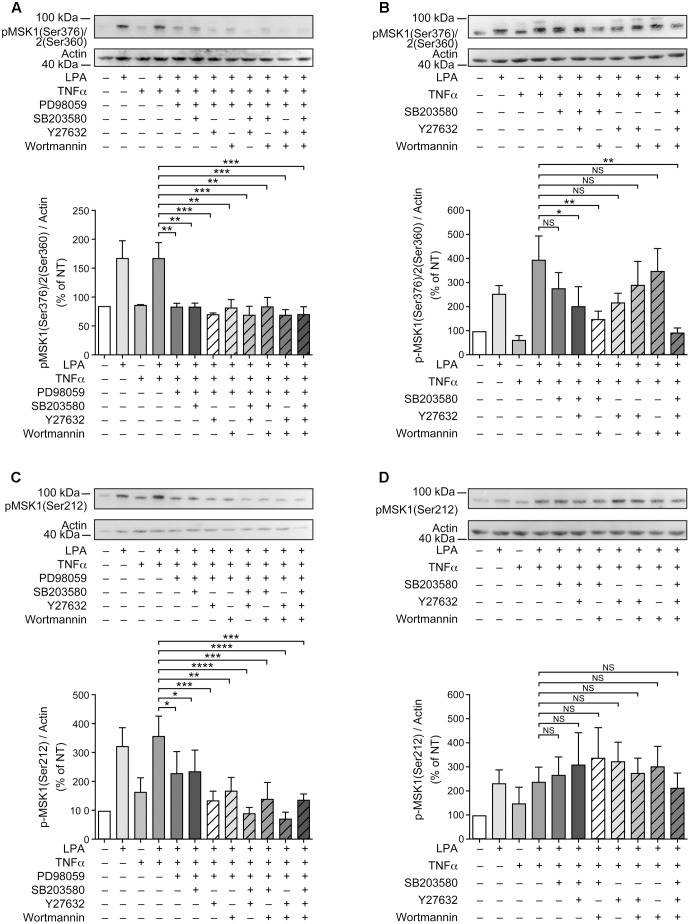
Combinations of signaling inhibitors attenuate LPA-mediated MSK phosphorylation. RAFLS were treated with or without TNFα (80 ng/ml) for 8 h before stimulation with 5 μM LPA for 5 min. Where indicated, cell samples were pre-treated for 30 min with the indicated combinations of inhibitors prior to stimulation with LPA. Inhibitor concentrations were 10 μM for PD98059, SB203580, and Y27632, and 200 nM for wortmannin. The levels of p-MSK1/MSK2 were monitored as described in Materials and Methods. Upper **(A–D)** are Western blots representative of at least 3 independent experiments with similar results. The lower panels are the densitometry quantification of p-MSK1 Ser376/MSK2 Ser-360 **(A,B)** and p-MSK1 Ser-212 **(C,D)** from 3 **(A–C)** or 6 **(D)** independent experiments. Protein bands were scanned and data were normalized with respect to actin as a loading control. The non-treated sample was set to 100% for comparison between experiments. Data are the mean value ± SEM. The resulting values were subjected to a one-way ANOVA, Dunnett’s multiple comparison test for selected groups. ^∗^*p* < 0.05; ^∗∗^*p* < 0.01; ^∗∗∗^*p* < 0.001; ^∗∗∗∗^*p* < 0.0001.

The next series of experiments evaluated the effects of inhibiting ERK in combination with one or more signaling pathways. Inhibition of both ERK and p38MAPK did not further reduce MSK phosphorylation compared to cells treated with ERK inhibitor alone (**Figures 5A,C**). Simultaneous inhibition of ERK signaling together with Rho kinase, or p38MAPK and Rho kinase, or p38MAPK and PI3K, or all of the signaling pathways together did not further reduce p-MSK1/2 (**Figure 5A**). In contrast, the same combinations of inhibitors slightly attenuated p-MSK1 phosphorylated at Ser-212 when compared to cells treated with the ERK inhibitor alone (**Figure 5C**). The most potent combinations were inhibition of ERK, p38MAPK, and Rho kinase, and that of ERK, Rho kinase, and PI3K signaling (**Figure 5C**).

Since inhibition of p38MAPK, Rho kinase, or PI3K alone partially inhibited LPA-induced p-MSK1/2 in TNFα primed RAFLS (**Figures 3A,B,D**, **5B**), we performed experiments to determine whether simultaneous inhibition of at least two pathways could further diminish MSK phosphorylation. As shown in **Figure 5B**, all combinations of inhibitors moderately decreased the levels of p-MSK1/2. However, inhibition of Rho kinase and p38MAPK or Rho kinase and PI3K did not decrease the levels of p-MSK1/2 compared to cells treated with the inhibitor of Rho kinase alone. Combination of p38MAPK and PI3K, or of p38MAPK, PI3K, and Rho kinase inhibitors reduced p-MSK1/2 to an extent greater than inhibition of either signaling pathway alone (**Figure 5B**). In contrast, these combinations of inhibitors did not reduce p-MSK1 at Ser-212 (**Figure 5D**). Taken together the data indicate that in both control and TNFα-primed RAFLS stimulation with LPA results in the phosphorylation of MSK1 at Ser-376 and Ser-212 and/or of MSK2 at Ser-360 via ERK. Although combination of p38MAPK and Rho kinase, p38MAPK and PI3K, or of p38MAPK, PI3K, and Rho kinase inhibitors reduced LPA-mediated phosphorylation event at Ser-376 of MSK1 (or Ser-360 of MSK2) in TNFα-primed cells, phosphorylation at Ser-212 of MSK1 was not inhibited.

### Signaling Pathways Involved in LPA-Mediated CREB Phosphorylation

**FIGURE 6:**
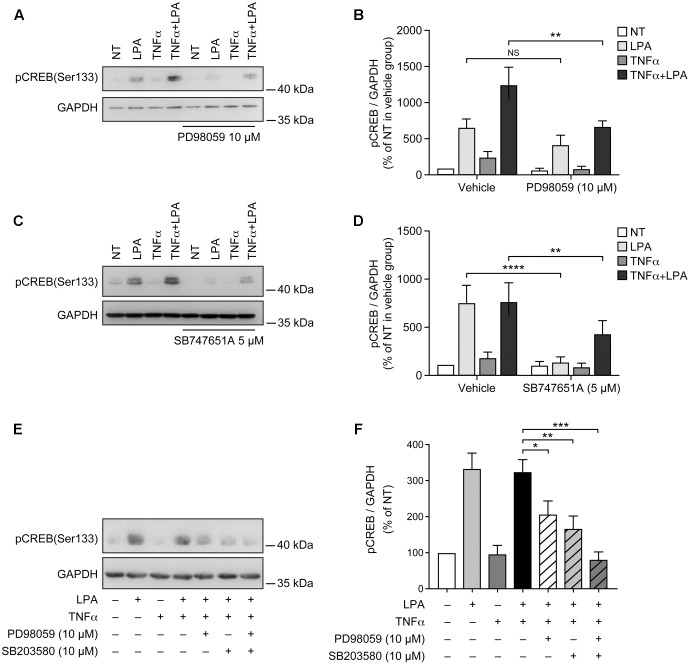
Lysophosphatidic acid-mediated CREB phosphorylation in TNFα-primed RAFLS is reduced by the inhibitors of ERK, p38MAPK, and MSKs. RAFLS were treated with or without TNFα (80 ng/ml) for 8 h before stimulation with 5 μM LPA for 5 min. Where indicated, cell samples were pre-treated for 30 min with the indicated inhibitors or their combination prior to stimulation with LPA. Left **(A,C,E)** are Western blots representative of at least three independent experiments with similar results. Right **(B,D,F)** are the densitometry quantification analysis. Data were normalized with respect to GAPDH as a loading control. The non-treated sample (NT) was set to 100% for comparison between experiments. Data are the mean value ± SEM. The resulting values in **(B,D)** were subjected to a two-way ANOVA, Sidak’s multiple comparison test. The values in F were subjected to a one-way ANOVA, Dunnett’s multiple comparison test for selected groups. ^∗^*p* < 0.05; ^∗∗^*p* < 0.01; ^∗∗∗^*p* < 0.001; ^∗∗∗∗^*p* < 0.0001.

LPA-mediated CREB phosphorylation at Ser-133 is MSK-dependent in RAFLS (Zhao et al., 2014). In the next series of experiments we assessed the impact of blocking MSK activity or the upstream MAPKs phosphorylation of MSK1/2 on LPA-mediated CREB phosphorylation in RAFLS. As anticipated the ERK inhibitor decreased LPA-mediated CREB phosphorylation by 41 ± 15% and 41 ± 11% (*p* < 0.01) in control and TNFα-primed cells, respectively (**Figures 6A,B**). Whereas the inhibitor of ERK or p38MAPK alone reduced LPA-mediated CREB phosphorylation by ∼50%, their combination completely blocked CREB phosphorylation in TNFα-primed cells (**Figures 6E,F**). The MSK inhibitor SB-747651A fully inhibited LPA-mediated CREB phosphorylation in unprimed cells and reduced the levels of phosphorylated CREB by 54 ± 4% (*p* < 0.01) in cells pre-treated with TNFα for 8 h (**Figures 6C,D**). Taken together, the data suggest that in both control and TNFα-primed cells LPA-induced CREB phosphorylation relies on p38MAPK and ERK signaling.

### Inhibition of p38MAPK, ERK, Rho Kinase, or MSK Activation Alone Does Not Regulate LPA-Mediated IL-8 Secretion in TNFα-Primed RAFLS

**FIGURE 7:**
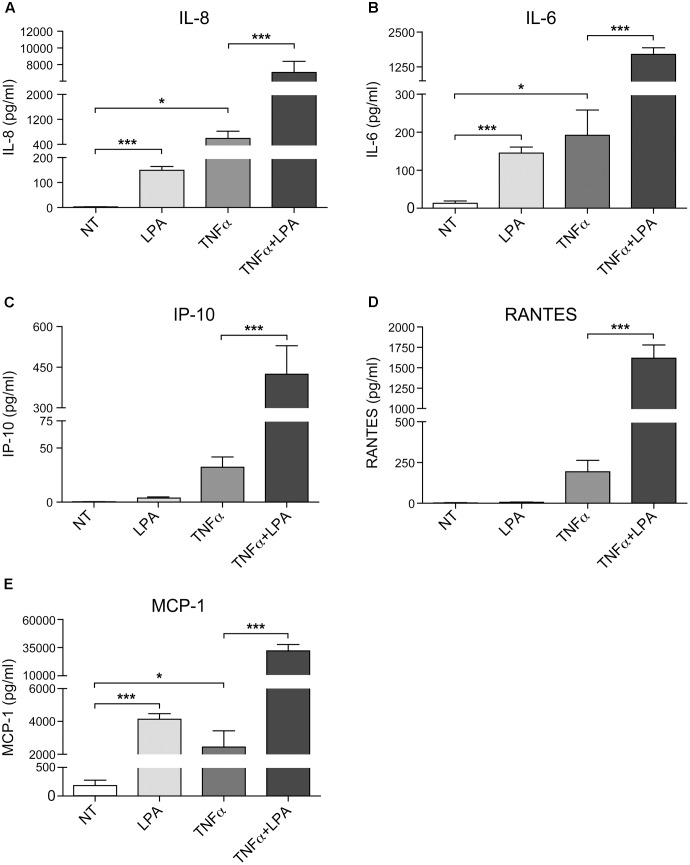
Priming of RAFLS with TNFα enhances LPA-mediated cytokine and chemokine secretion. RAFLS were treated with or without TNFα (80 ng/ml) for 8 h before stimulation with 5 μM LPA for 24 h. The supernatants were collected and the levels of IL-8 **(A)**, IL-6 **(B)**, IP-10 **(C)**, RANTES **(D)**, and MCP-1 **(E)** were monitored using a Luminex immunoassay as described in “Materials and Methods.” Data are the mean value ± SEM from at least three independent experiments. The values were subjected to Student’s *t*-test. ^∗^*p* < 0.05; ^∗∗^*p* < 0.01; ^∗∗∗^*p* < 0.001.

**FIGURE 8:**
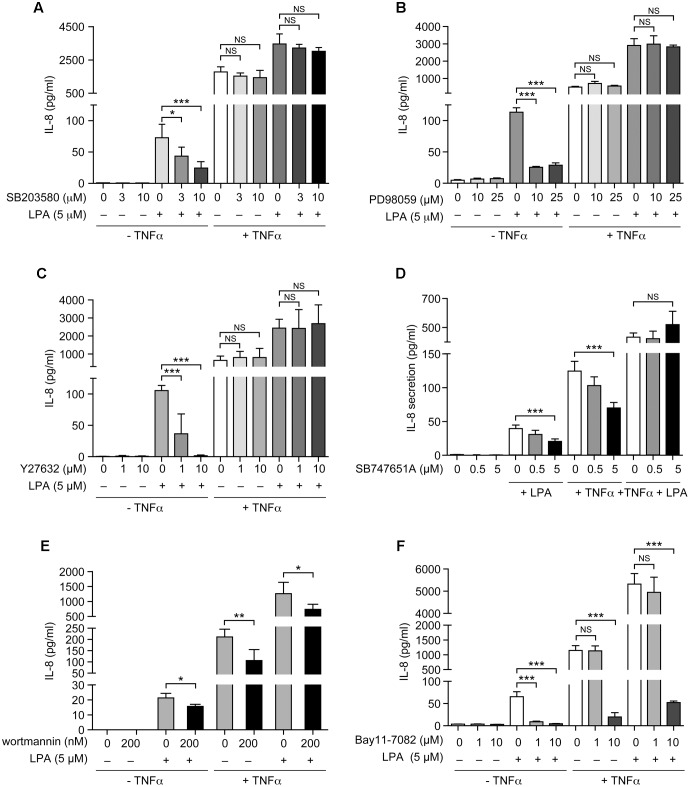
The sensitivity of LPA-mediated IL-8 secretion to signaling inhibitors is altered after priming of RAFLS with TNFα. RAFLS were treated with or without TNFα (80 ng/ml) for 8 h before stimulation with 5 μM LPA for 24 h. Where indicated, cell samples were pre-treated for 30 min with the indicated concentrations of the p38MAPK **(A)**, ERK1/2 **(B)**, Rho kinase **(C)**, MSK **(D)**, PI3K **(E)**, or NF-κB **(F)** inhibitor prior to stimulation with LPA. The supernatants were collected and the IL-8 ELISA was performed. Data are the mean value ± SEM (pg/ml) from 3 **(B–D,F)** or 6 **(E)** independent experiments. The values were subjected to Student’s *t*-test. ^∗^*p* < 0.05; ^∗∗^*p* < 0.01; ^∗∗∗^*p* < 0.001.

As show in **Figure 7** and as previously reported, priming of RAFLS with TNFα for 8 h strongly enhances the subsequent release of IL-8 as well as of other cytokines/chemokines in response to stimulation with LPA (Zhao et al., 2008, 2014). Early reports also highlighted that this super-production of cytokines/chemokines by TNFα-conditioned cells was dependent on LPA_1_ and LPA_3_ receptor activation (Zhao et al., 2008). In TNFα-primed cells, selective LPA_1/3_ antagonist inhibited the super-production of IL-8 (and IL-6 as well) induced by LPA (Zhao et al., 2008). TNFα-primed air pouch tissues in LPA_3_ knockout mice release less cytokines/chemokines in response to LPA when compared to control mice, and treatment with a selective LPA_1/3_ antagonist was necessary to totally block cytokine/chemokine production in this mouse model of inflammation (Zhao et al., 2011). Though LPA was reported to induce cytokine/chemokine secretion through activation of multiple signaling pathways including MSK1, MSK2, ERK, and p38MAPK (Zhao et al., 2008, 2014), the contribution of MSKs and other kinase cascades to LPA-mediated cytokine/chemokine production after priming with TNFα has not been investigated. In the next series of experiments we investigated in primed and unprimed cells the impact of inhibiting p38MAPK, ERK, Rho kinase, PI3K, MSKs, or the transcription factor NF-κB on LPA-mediated IL-8 secretion. Consistent with previous studies, inhibition of p38MAPK, ERK, Rho kinase, or MSKs significantly reduced LPA-mediated IL-8 synthesis (**Figures 8A–D**). Inhibition of PI3K and NF-κB also decreased LPA-induced IL-8 secretion (**Figures 8E,F**).

IL-8 secretion due to TNFα priming was sensitive to inhibition by SB-747651A (**Figure 8D**), wortmannin (**Figure 8E**), and the highest dose of Bay11-7082 (**Figure 8F**), whereas the other signaling inhibitors were without effect. However, control experiments revealed that the effect of the NF-κB inhibitor (Bay11-7082) at 10 μM was cytotoxic for RAFLS whereas all other compounds had no significant impact on cell viability (Supplementary Figures 2, 3). Surprisingly, LPA-induced IL-8 secretion became insensitive to the inhibitors of p38MAPK, ERK, Rho Kinase, MSKs, and NF-κB in TNFα-primed cells (**Figures 8A–D,F**). Only LPA-induced IL-8 secretion remained sensitive to inhibition by wortmannin after priming with TNFα (**Figure 8E**).

### Combination of Signaling Inhibitors Can Reduce LPA-Induced IL-8 Secretion after TNFα Priming

**FIGURE 9:**
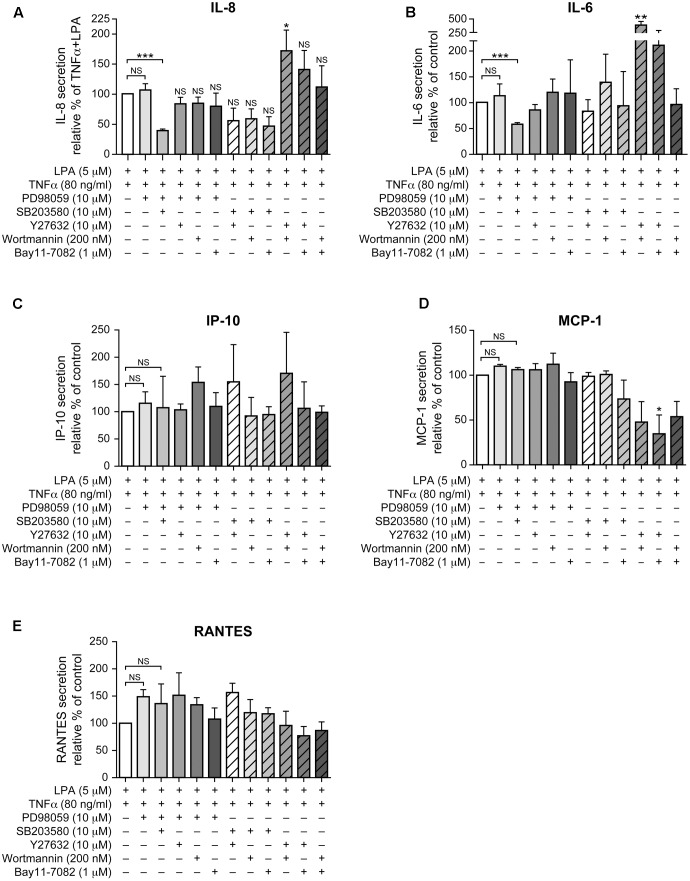
Combinations of signaling inhibitors differentially impact LPA-mediated IL-8, IL-6, IP-10, MCP-1 and RANTES secretion in TNFα-primed cells. RAFLS were treated with or without TNFα (80 ng/ml) for 8 h before stimulation with 5 μM LPA for 24 h. Where indicated, cells were pre-treated for 30 min with the indicated concentrations of the p38MAPK **(A)**, ERK1/2 **(B)**, Rho kinase **(C)**, MSK **(D)**, PI3K **(E)**, or NF-kB (F) inhibitor prior to stimulation with LPA. The supernatants were collected and the levels of IL-8 **(A)**, IL-6 **(B)**, IP-10 **(C)**, MCP-1 **(D)**, and RANTES **(E)** were monitored using a Luminex immunoassay as described in “Materials and Methods.” The level of cytokine/chemokine produced by TNFα-primed RAFLS stimulated with LPA was set to 100% for comparison between experiments. Data are the mean value ± SEM from 4 **(A,B,E)** or 3 **(C,D)** independent experiments. The values were subjected to a one-way ANOVA, Dunnett’s multiple comparison test. ^∗^*p* < 0.05; ^∗∗^*p* < 0.01; ^∗∗∗^*p* < 0.001.

As neither the p38MAPK, ERK, Rho kinase, nor the NF-κB inhibitors (1 μM) acting independently could inhibit IL-8 in response to LPA in TNFα-primed cells, we next tested various combinations of signaling inhibitors at concentrations that had no significant impact on cell viability (Supplementary Figure 3). The combined inhibition of p38MAPK and ERK that completely blocked the MSK-CREB signaling axis (**Figures 5A,C**, **6E,F**) significantly decreased the production of IL-8 by 61 ± 3% (**Figure 9A**) when compared to TNFα-primed RAFLS stimulated with LPA. Though not statistically significant, combinations of the p38MAPK inhibitor with those of Rho kinase, PI3K, or NF-κB showed a tendency to more strongly decrease IL-8 secretion compared to other combinations of inhibitors (**Figure 9A**), thereby suggesting a role for p38MAPK in exacerbating LPA-induced IL-8 release in TNFα-primed RAFLS. In contrast, combined inhibition of Rho kinase and PI3K or of Rho kinase and NF-κB increased the release of IL-8 by 72.5% (p < 0.05) and 40 ± 30%, respectively (**Figure 9A**).

The pattern of IL-6 secretion by cells treated with the same combinations of signaling inhibitors was similar to that of IL-8. LPA-induced IL-6 secretion after TNFα priming, which was not inhibited by a pre-incubation with either the inhibitor of p38MAPK (not shown) or of ERK (**Figure 9B**), was reduced by 41 ± 3% when these two pathways were inhibited (**Figure 9B**). Combination of Rho kinase and PI3K inhibitors significantly enhanced (260%, *p* < 0.01) the release of IL-6 compared to the control TNFα-primed cells stimulated with LPA. MCP-1, IP-10, and RANTES exhibited different inhibition patterns with combinations of inhibitors. The release of IP-10 and of RANTES was not affected by any of the combinations of inhibitors (**Figures 9C,E**) whereas combinations of Rho kinase and PI3K, Rho kinase and NF-κB, or PI3K and NF-κB inhibitors decreased MCP-1 secretion to 48 ± 23%, 34 ± 21%, and 53 ± 17% of control LPA-stimulated cells, respectively (**Figure 9D**).

## Discussion

Lysophosphatidic acid is a pleiotropic lipid growth factor that regulates the various functional responses of RAFLS including secretion of IL-8 and IL-6 by signaling through ERK, p38MAPK, and Rho kinase (Zhao et al., 2008). Consistent with a previous study, IL-8, IL-6, IP-10, MCP-1 and RANTES secretion mediated by LPA was strongly enhanced by TNFα priming (Zhao et al., 2014). Dual LPA1/3 receptor antagonists block LPA-mediated super-production of cytokines/chemokines after preconditioning of cells with TNFα (Zhao et al., 2008). LPA_1_ and LPA_3_ receptors may contribute to cytokine/chemokine super-production. Targeting LPA receptors, in particular LPA_1_, have been proposed as a treatment for various diseases including RA (Orosa et al., 2015). LPA_3_ expression is up-regulated by TNFα in RAFLS (Zhao et al., 2008). In other cell lines, LPA_3_ receptors are known to couple to Gαi/o proteins to activate PI3K and Ras-MAPK signaling (Ishii et al., 2000). This study shows that LPA-induced IL-8 secretion can be inhibited by inhibitors of p38MAPK, ERK, MSK and PI3K possibly through LPA_1_ and LPA_3_ receptor-dependent activation of Gαi/o proteins. Except for PI3K, we show that after cell priming with TNFα, LPA-mediated IL-8 secretion becomes insensitive to inhibition of p38MAPK, ERK, Rho kinase, MSK and NF-κB as well. CREB is a transcription factor known to regulate the expression of multiple gene and various physiological processes (Steven and Seliger, 2016). Although MSK and ERK1/2 inhibitors inhibited MSK-mediated phosphorylation of CREB, LPA-mediated IL-8 production in TNFα-primed RAFLS was not reduced. The data highlight that concomitant inhibition of the ERK-MSK-CREB signaling axis and that of p38MAPK pathways was required to selectively and significantly reduce LPA-induced IL-8 and IL-6 secretion in TNFα-treated cells.

**FIGURE 10:**
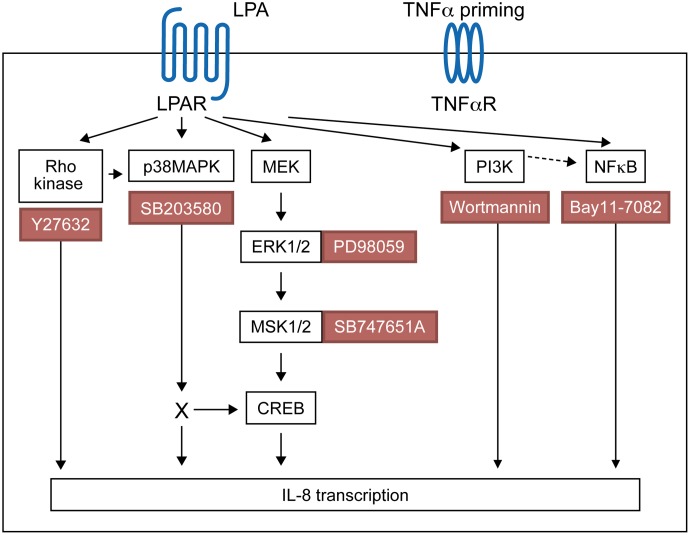
Proposed signaling pathways involved in LPA-induced IL-8 secretion in TNFα-primed RAFLS.

Using pharmacological inhibitors or siRNA, we previously demonstrated that LPA induces MSK1 and MSK2 activation and MSK-mediated CREB phosphorylation at Ser-133, thereby promoting the synthesis of IL-8 and MCP-1 (Zhao et al., 2014). Here we show that MSK phosphorylation and CREB phosphorylation in response to LPA is reduced by an inhibitor of ERK activation. Though LPA induces activation of p38MAPK (Zhao et al., 2008), our data indicate that p38MAPK does not significantly contribute to MSK1 or MSK2 activation. There are four differentially expressed p38MAPK isoforms (α, β, γ, and δ) in tissues, with only p38MAPKα and p38MAPKβ being sensitive to inhibition by SB203580 (Cuenda and Rousseau, 2007). Using fibroblasts from knockout mice, it has been shown that p38MAPKα, but not p38MAPKβ, phosphorylates MSKs (Beardmore et al., 2005; Darragh et al., 2005). Though all p38MAPK isoforms are expressed within the RA synovium, α and γ isoforms were preferentially activated in those inflamed tissues (Korb et al., 2006). Furthermore expression of p38MAPK isoforms was cell specific with p38MAPKβ and p38MAPKγ being preferentially expressed by RAFLS (Korb et al., 2006). The fact that RAFLS do not express the p38MAPKα isoform involved in MSK activation may explain why SB203580 does not prevent LPA-induced MSK phosphorylation. In this study we established that LPA activates ERK-MSK signaling, which in turn phosphorylates CREB (Zhao et al., 2014), and ultimately induces IL-8 secretion by RAFLS. Activation of p38MAPK, Rho kinase, or PI3K contributes to LPA-induced IL-8 secretion but through MSK-independent and possibly CREB-dependent signaling pathways (**Figure 10**).

Regulation of IL-8 expression involves kinase cascades and transcription factors such as AP-1, CREB, C/EBP-1, and NF-κB that bind to the IL-8 promoter (Hoffmann et al., 2002; Saatian et al., 2006; Mayer et al., 2013; Zhao et al., 2014). Interestingly, inhibition of ERK and p38MAPK signaling was the only combination that significantly reduced LPA-induced IL-8 secretion in TNFα-primed RAFLS. The production of IL-6 but not that of other chemokines (MCP-1, RANTES, IP-10) was also reduced by this combination of inhibitors, thereby suggesting the involvement of similar signaling networks and transcription factors in IL-8 and IL-6 gene expression. Targeting p38MAPK or ERK as a potential therapeutic target for treatment of RA has been considered (Ohori, 2008; Arthur and Ley, 2013). Though potent and isoform-specific inhibitors of p38MAPK have been generated, most phase 2 clinical trials have failed due to very limited improvement of RA symptoms or because the drug was not well tolerated (Damjanov et al., 2009; Sweeney, 2009). Inhibition of cytokine/chemokine synthesis by RAFLS by signaling inhibitors, including those of MAPK pathways, depends on how cells are activated (Jones et al., 2017). A drug targeting transforming growth-factor-β-activated kinase-1, a master kinase upstream of NF-kB, JNK, and p38MAPK, as well MEK1/2 was able to inhibit the production of cytokines/chemokines by TNFα-stimulated cells, whereas selective inhibitors of MEK1/2 and of p38MAPK had little impact (Jones et al., 2017). Our study suggests that in chronic inflammatory diseases inhibition of signaling through both p38MAPK and ERK1/2 would be required to dampen the vicious cycle of inflammation.

RAFLS are characterized by the ability to synthesize various inflammatory cytokines/chemokines with immune regulatory properties (Bottini and Firestein, 2013). Blocking the functions of cytokines such as TNFα or IL-6 reduces joint damage progression in RA patients (Smolen et al., 2012). TNFα induces a transient activation of MSK1 and MSK2 in keratinocytes (Seidel et al., 2011) and in RAFLS as well (Zhao et al., 2014). Phosphorylation of MSK1/2 (Ser-372 or Ser-360) returns to basal levels after addition of TNFα for 8 h (**Figure 1A**). Surprisingly ERK-dependent phosphorylation of MSK1 at Ser-212 remained elevated compared to unstimulated RAFLS. In TNFα-primed RAFLS, LPA consistently enhanced p-MSK1/2 and p-MSK1 at Ser-212 mainly via ERK activation. After priming with TNFα, inhibition of p38MAPK, Rho kinase, and PI3K slightly reduced LPA-mediated p-MSK1/2 without reducing MSK1 phosphorylation at Ser-212 (**Figures 3D**, **4D**), suggesting that p38MAPK, Rho kinase and PI3K may regulate MSK2. Furthermore, combinations of inhibitors that further decreased the levels of p-MSK1/2 had no impact on p-MSK1 at Ser-212. MSK1 activation by TNFα and IL-1β has been reported in human keratinocytes. These studies report that a non-specific inhibitor of NF-κB reduced MSK1 autophosphorylation at Ser-376, but had no impact on the sites (Ser-360 and Thr-581) directly phosphorylated by ERK or p38MAPK (Gesser et al., 2007; Seidel et al., 2011). The mechanisms by which p38MAPK, Rho kinase, and PI3K specifically modulate p-MSK remain to be investigated. Reduction of MSK1 autophosphorylation at Ser-376 is unlikely to be secondary to inhibition of the C-terminal kinase domain of MSK1 by p38MAPK, Rho kinase, or PI3K inhibitors, since autophosphorylation of Ser-212 is not reduced (Deak et al., 1998; McCoy et al., 2005). It remains to be determined whether MSK1 and MSK2 activation in RAFLS involves distinct kinase cascades. Whereas activation of MSKs relies on ERK, it cannot be excluded that dephosphorylation of MSK1 at Ser-212 by unknown phosphatases is delayed compared to Ser-376. More careful analysis of the kinetics of MSK dephosphorylation and identification of the phosphatases involved would be required.

Class I PI3Kδ expressed in the RA synovial intimal lining was reported to regulate FLS growth and TNFα signaling (Bartok et al., 2014). The severity of inflammation in animal models of RA is reduced by PI3Kγ inhibitors or in mice knockout for PI3Kγ (Camps et al., 2005; Hawkins and Stephens, 2015). TNFα and LPA activate PI3K signaling (Malemud, 2015; Riaz et al., 2016). Consistent with this, inhibition of PI3K signaling using the pan-PI3K inhibitor wortmannin, a small fungal metabolite targeting the p110 subunit of PI3K (El-Kholy et al., 2003), partially reduced LPA-induced IL-8 secretion in unprimed and TNFα-primed RAFLS. The mechanisms by which wortmannin reduces IL-8 production were not investigated. Nevertheless LPA-mediated p-MSK1/2 in TNFα primed cells was reduced by wortmannin (**Figure 3D**). We cannot totally exclude that inhibition of PI3K could interfere indirectly with Rho kinase, ERK and/or p38MAPK activation (Hawkins and Stephens, 2015), thereby reducing the MSK-dependent and independent pathways involved in cytokine/chemokine production.

LPA and TNFα are well known to activate NF-κB signaling in various cell types including synovial fibroblasts (Sun and Yang, 2010; Brenner et al., 2015). Upon activation with TNFα, NF-κB becomes phosphorylated on Ser-536 by the IKK kinase complex (Brenner et al., 2015). NF-κB is also a MSK substrate (Reber et al., 2009; Lin et al., 2015; Terazawa et al., 2015). Inhibition or silencing of MSK1 was reported to decrease NF-κB Ser-276 phosphorylation and was linked to expression of stem cell factor in human lung fibroblasts (Reber et al., 2009) and to IL-8 in epithelial cells (Lin et al., 2015). LPA-induced NF-κB signaling may contribute to cytokine/chemokine synthesis by RAFLS, since inhibition of NF-κB significantly decreased IL-8 secretion induced by LPA alone, whereas combined inhibition of NF-κB and p38MAPK, and of NF-κB and Rho kinase attenuated LPA-induced IL-8 and MCP-1 production in TNFα-primed RAFLS. However, in contrast to RAFLS preconditioned with TNFα, we did not observe degradation of IkB or phosphorylation of NF-κB at Ser-536 in cells stimulated with LPA alone, whereas stimulation of TNFα-primed RAFLS with LPA attenuated NF-κB Ser-536 phosphorylation (unpublished data). A drawback is that the NF-κB inhibitor (Bay11-7082) does not inhibit the IKKs, but suppresses their activation by targeting components of the ubiquitin system (Strickson et al., 2013), and has possible off-target effects (Supplementary Figures 2, 3). Complementary approaches using the MSK inhibitor SB-747651A or silencing of MSKs in RAFLS will be required to determine whether MSK phosphorylates NF-κB at Ser-276 in control and TNFα-primed RAFLS stimulated with LPA.

In this study we show that LPA-induced IL-8 production is mediated through the ERK1/2-MSK signaling axis and MSK-independent pathways. A summary of the proposed signaling pathways involved in LPA-induced IL-8 production in TNFα-primed RAFLS has been presented in **Figure 10**. Priming of RAFLS with TNFα for 8 h enhances LPA-mediated cell functional responses including MSK activation and cytokine/chemokine production. However, after inflammatory conditioning with TNFα, LPA-mediated cytokine/chemokine secretion was mainly MSK-independent and insensitive to inhibition by Rho-kinase, p38MAPK, or NF-κB inhibitor.

The key finding is that simultaneous inhibition of at least two signaling pathways such as ERK1/2 and p38MAPK was required to inhibit IL-8 and IL-6 production in TNFα-preconditioned RAFLS. Since both TNFα and LPA activate p38MAPK and ERK-MSK-CREB signaling, drug combinations targeting these two pathways are potential new strategies to treat RA.

## Author Contributions

WH, CZ, and SB designed the experiments. WH and CZ performed the experiments and analyzed the data. WH and SB wrote the paper.

## Conflict of Interest Statement

The authors declare that the research was conducted in the absence of any commercial or financial relationships that could be construed as a potential conflict of interest. The reviewer MP and handling Editor declared their shared affiliation.

## Abbreviations

ATX: autotaxin
DMEM: Dulbecco’s modified Eagle medium
ERK: extracellular signal–regulated kinases, also named p42/44 MAPK
FBS: fetal bovine serum
FLS: fibroblast-like synoviocytes
IL-6: interleukin-6
IL-8: interleukin-8, also named CXCL8
IP-10: IFN-γ inducible Protein 10 (also named CXCL10)
LPA: lysophosphatidic acid
LPC: lysophosphatidylcholine
MAPK: mitogen activated protein kinase
MCP-1: monocyte chemoattractant protein, also named CCL2
MSK: mitogen- and stress-activated kinase
p-MSK1: phosphorylation of MSK1 at Ser-212
p-MSK1/2: phosphorylation of MSK1 at Ser-376 and/or MSK2 at Ser-360
RA: rheumatoid arthritis
RANTES: Regulated on Activation, Normal T-cell Expressed and Secreted, also named CCL5
ROCK: Rho kinase or Rho-associated protein kinase
TNF α: tumor necrosis factor α
