# Supplementary material for: Differential Effects of Inhibitor Combinations on Lysophosphatidic Acid-Mediated Chemokine Secretion in Unprimed and Tumor Necrosis Factor-α-Primed Synovial Fibroblasts
Source: Front Pharmacol. 2017 Nov 21;8:848. doi: 10.3389/fphar.2017.00848 (PMC5702485; doi:10.3389/fphar.2017.00848)

Supplementary material

Supplementary Figure 1: Schematic of experiment design.

Supplementary Figure 2: High concentrations of Bay11-7082 totally block LPA-induced MSK phosphorylation but are highly cytotoxic for RAFLS. (A) RAFLS cultured in 24-well plates were starved for 16 h in FBS-free DMEM prior to treatment with 10 μM Bay11-7032 for 24 h. Cells were washed with PBS and treated with Accutase® Cell Detachment Solution at room temperature for 10 min. Reactions were stopped by adding DMEM supplemented with 10% FBS. Cells were collected by centrifugation, washed once in PBS, and suspended in PBS for staining with propidium iodine (PI) and Annexin V-eFluor450 (BD Pharmingen, Oakville, ON, Canada) for 20 min. Labeled cells were then analyzed by flow cytometry (Q1: PI+ Annexin V-; Q2: PI+ Annexin V+; Q3: PI- Annexin V-; Q4, PI- Annexin V+). (B) Cells were treated with or without TNFα (80 ng/ml) for 8 h before stimulation with 5 μM LPA for 5 min. Where indicated the cells were pre-treated for 30 min with 10 μM Bay11-7032 prior to stimulation with LPA. The levels of p-MSK1 Ser-376/MSK2 Ser-360 were monitored as described in Materials and Methods. The upper panel is a blot representative of three independent experiments with similar results. The lower panel is the densitometry quantification of p-MSK1 Ser376/MSK2 Ser-360. Data were normalized with respect to actin as a loading control. The non-treated (NT) sample was set to 100% for comparison between experiments. Data are the mean value ± SEM. Data were subjected to a two-way ANOVA, with Sidak's multiple comparison test. ** $p<0.01$.

Supplementary Figure 3: Cytotoxicity of different inhibitors or combinations of these inhibitors. RAFLS cultured in 24-well plates were starved for 16 h in FBS-free DMEM and treated with the indicated concentrations of inhibitors for 24 h. Cells were washed with PBS and treated with Accutase® Cell Detachment Solution at room temperature for 10 min. Reactions were stopped by adding DMEM supplemented with 10% FBS. Cells were collected by centrifugation, washed once in PBS, and suspended in PBS for staining with propidium iodine (PI). Labeled cells were then analyzed by flow cytometry as in supplementary figure 2.

Supplementary Figure 1

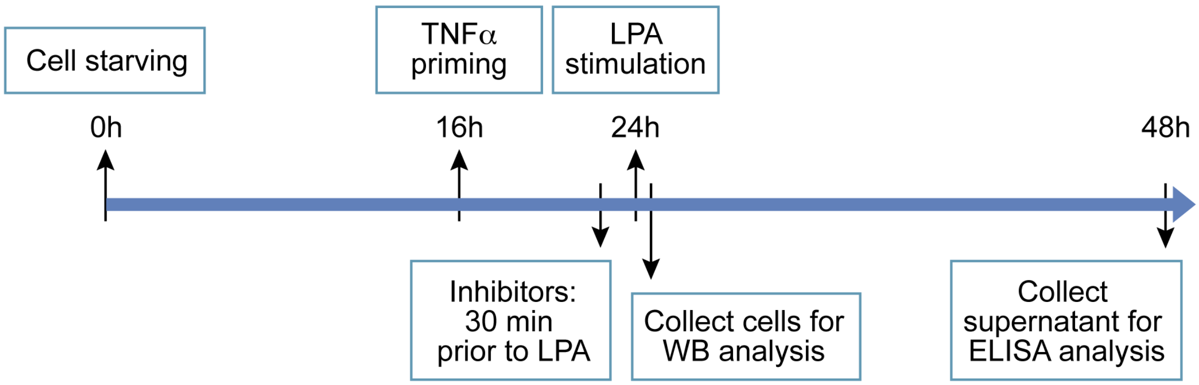

Supplementary Figure 2

A

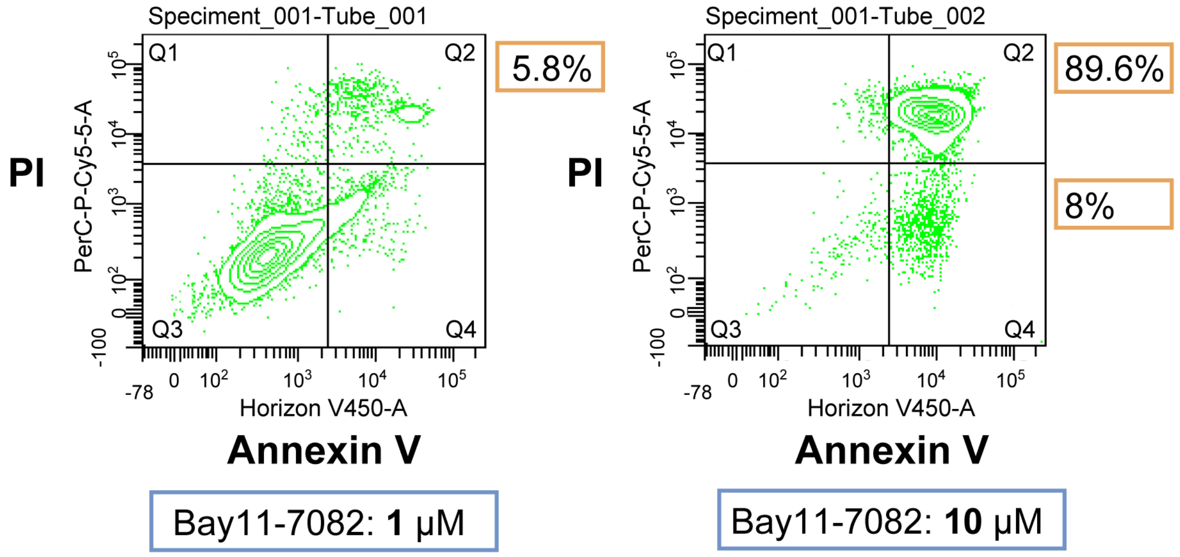

B

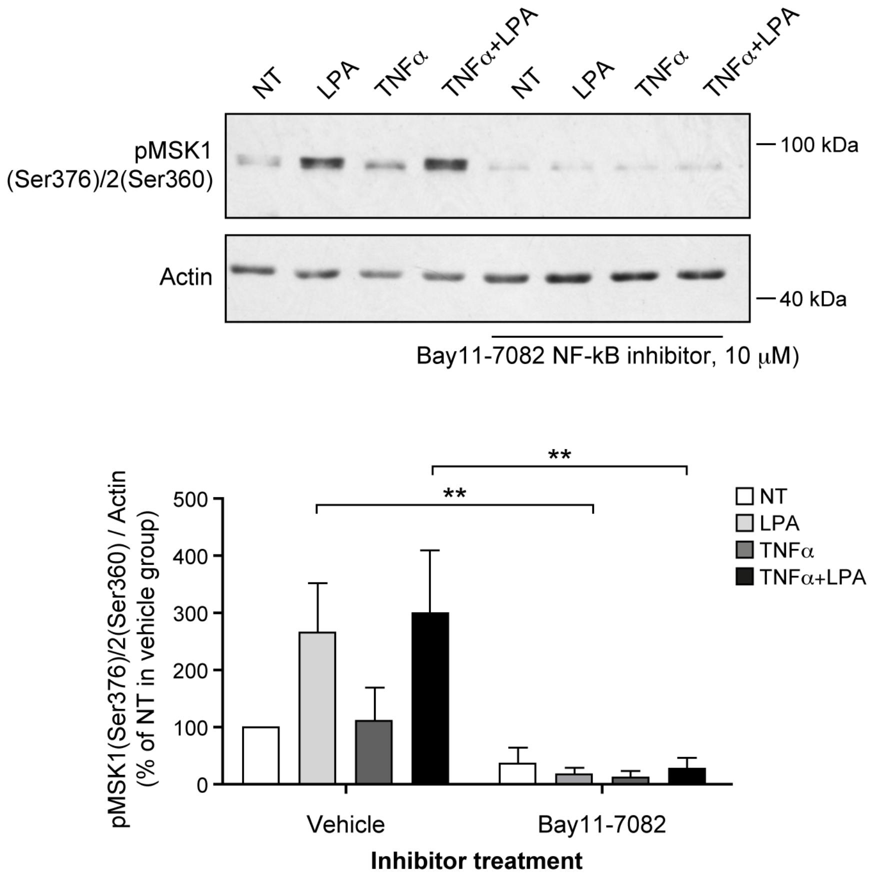

Supplementary Figure 3

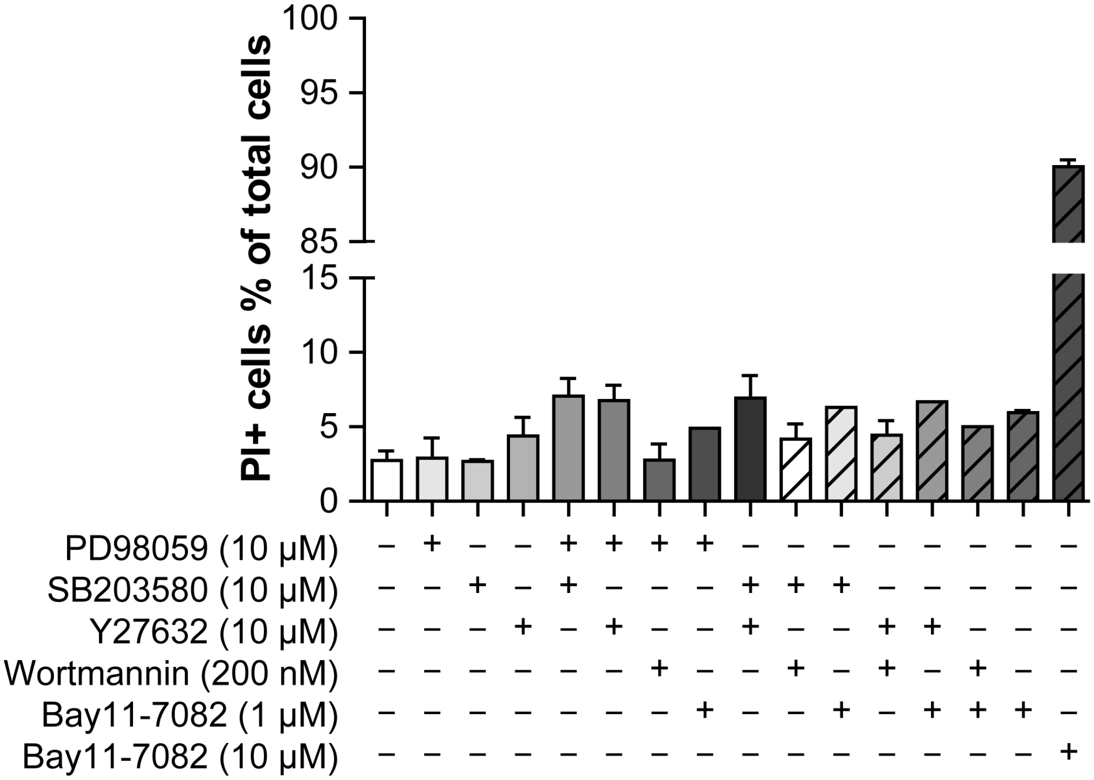

Supplement: Supplementary file 1 [file Presentation_1.PDF]
